# Recent advances in gold nanoparticle-graphene hybrid nanoplatforms with visible to near-infrared response for photodynamic and photothermal therapy and bioimaging

**DOI:** 10.1039/d4ra09100k

**Published:** 2025-04-15

**Authors:** Alexandru Holca, Vlad Cucuiet, Simion Astilean, Marc Lamy de la Chapelle, Monica Focsan

**Affiliations:** a Department of Biomolecular Physics, Faculty of Physics, Babes-Bolyai University M. Kogalniceanu 1 400084 Cluj-Napoca Romania monica.iosin@ubbcluj.ro; b Nanobiophotonics and Laser Microspectroscopy Center, Interdisciplinary Research Institute in Bio-Nano-Sciences, Babes-Bolyai University T. Laurian 42 400271 Cluj-Napoca Romania; c Le Mans Institute of Molecules and Materials (IMMM – UMR6283), Le Mans University Avenue Olivier Messiaen Le Mans 72085 Cedex 9 France marc.lamydelachapelle@univ-lemans.fr

## Abstract

Photodynamic therapy (PDT) and photothermal therapy (PTT) are light-activated cancer treatments. PDT involves the administration of a photosensitizing agent, which is activated by light of a specific wavelength to generate reactive oxygen species. Alternatively, PTT involves the use of photothermal agents, which are materials that absorb light and convert it into heat. Gold nanoparticles are often used as photothermal agents owing to their localized surface plasmon resonance (LSPR), a key optical property, which allows them to efficiently absorb light and convert it into heat. Graphene, which is a 2D material with extraordinary optical and physical properties and a large surface area, shows great promise both in PDT and PTT as an intrinsic nanoheater or a versatile platform for the immobilization of gold nanoparticles and other functional molecules, including photosensitizers. Moreover, graphene-based derivatives, *i.e.* graphene oxide (GO) and reduced graphene oxide (rGO), exhibit intrinsic optical/spectroscopic signals, which can be used in fluorescence, Raman and thermal imaging. By combining gold nanoparticles with graphene derivatives, a higher increase in temperature can be achieved under light irradiation owing to the synergistic effect of these two materials and the drug delivery efficiency and multimodal imaging techniques can be enhanced. This review provides insights into graphene-based nanoplatforms, focusing on multimodal therapy and imaging techniques. Furthermore, future perspectives in the field of graphene-based- and hybrid-nanoplatforms are suggested.

## Abstract 2

Graphene oxide (GO) and reduced graphene oxide (rGO) with gold nanoparticles (GNPs) enhance phototherapy, leveraging the NIR-I and NIR-II optical biological windows for improved tissue penetration and targeted cancer treatment.

## Graphical abstract

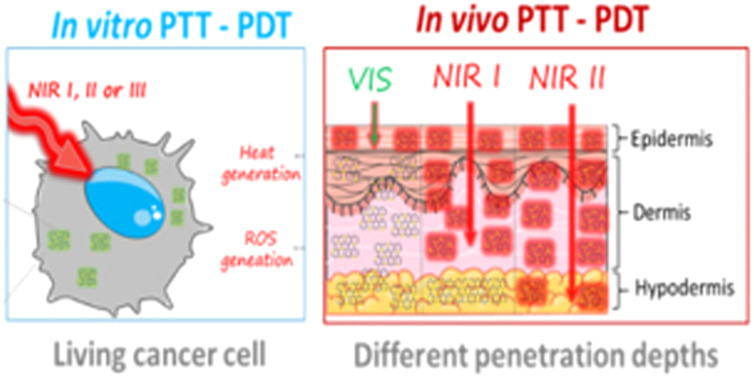

## 1. Introduction

**Fig. 1:**
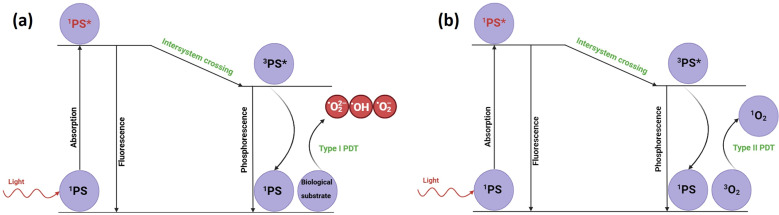
Schematic of type I PDT (a) and type II PDT (b).

Cancer is one of the leading causes of death worldwide.^1^ Owing to the high incidence number amongst various populations, several drugs have been developed to treat this disease.^2^ However, some of these drugs do not specifically target cancer cells. Following this issue, other types of therapies have been designed and tested to treat cancer, such as immunotherapy,^3^ photodynamic therapy (PDT)^4^ and photothermal therapy (PTT).^5^ Generally, PDT and PTT use nanoparticles, molecules and light to induce apoptosis or necrosis in cancer cells through different mechanisms. PDT requires three factors, *i.e.*, the presence of a photosensitizer (PS), oxygen and irradiation with NIR light (650–1350 nm),^6^ and it can be described as the generation of reactive oxygen species (ROS) (*i.e.* superoxide, peroxide and hydroxyl radicals) as result of PS in the presence of light. ROS can be generated through two mechanisms of PDT. Generally, the NIR region is also called the biological transparency window owing to the low absorption and scattering in the tissue. Moreover, as light goes from 650 nm to 1350 nm, its penetration into tissues increases.^7^ The PS exists in the ground state as ^1^PS, and when further excited with a light source, it absorbs a photon, thus gaining energy and converting to ^1^PS*. Following that, ^1^PS* undergoes non-radiative processes, such as vibrational relaxation and internal conversion, reaching the lowest vibrational level of the excited state. From the lowest level of the excited state, ^1^PS* emits a photon with a lower energy compared with that of the absorbed photon, which is a phenomenon called fluorescence. However, the fluorescence lifetime is in the range of nanoseconds, while by intersystem crossing to the triplet state (^3^PS*) and radiative emission, the phosphorescence phenomenon is observed, which has a lifetime in the range of microseconds. Moreover, ^3^PS* can participate in energy transfer. In the type I photoreaction (Fig. 1a), the PS in the excited state reacts with the biological substrate and oxygen to generate ROS radicals (superoxide, ˙O_2_^−^; peroxide, ˙O_2_^−2^; and hydroxyl radicals, HO^−^).^6,8^ Alternatively, the type II photoreaction (Fig. 1b) generates singlet oxygen (^1^O_2_) by directly transferring energy from the triplet state of the PS, ^3^PS*, to molecular oxygen, O_2_, which is abundantly present in its triplet state, ^3^O_2_. The singlet oxygen state is highly reactive and can induce oxidative stress in cells,^9^ resulting in cell death through apoptosis, necrosis or autophagy.^6^

The PS should present several properties to enhance its PDT efficiency and lower its side-effects, such as low dark toxicity, target the affected tissue, good transport and spreading through the blood, selectivity for binding with the tumor,^10^ high fluorescence quantum yield due to its higher emission efficiency, which translates to better excitation of the ^3^PS*, and fast elimination from the organism. The PS is internalized in tumor cells through different mechanisms such as endocytosis or based on the hydrophilicity of the PS.^11^ Basically, hydrophobic PS can be internalized by the diffusion mechanism through the plasma membrane, while hydrophilic PS are internalized through endocytosis.

Depending on the PS, PDT has different advantages and disadvantages. The first generation of PSs, such as hematoporphyrin derivatives, has a short wavelength maximum absorption and its elimination time from organisms is long, thus causing skin hypersensitivity when the skin is exposed to light. The second generation of PSs, for example chlorin, presents a better maximum absorption wavelength in the first NIR region (650–800 nm). Also, its elimination time is shorter, presenting higher selectivity for cancer cells. The third generation of PSs is highly selective due to the derivatization of the second generation of PSs with specific target molecules for the receptors of cancer cells.^12^

Compared to PDT, PTT uses photothermal agents, which are also excited when irradiated with specific light sources. However, instead of the intersystem crossing mechanism, which produces ROS, vibrational relaxation induces heat generation in PTT.

**Fig. 2:**
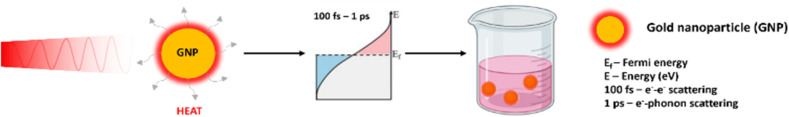
Schematic of hot-electron excitation and heat generation.

Metal nanoparticles exhibit localized surface plasmon resonance (LSPR). When nanoparticles are irradiated with light, the free electrons localized inside them oscillate collectively with the same frequency. Briefly, the heating mechanism involves electron–phonon relaxation and phonon–phonon relaxation.^13^ This relaxation process occurring on an ultra-short timescale is followed by thermal energy exchange through the nanoparticle surface toward the surrounding medium, which is strongly dependent on the heat capacity and thermal conductance of the surrounding medium. Under light irradiation, the electrons on the surface of gold nanoparticles (GNPs) are excited and are referred to as hot electrons when their energy exceeds the Fermi level. During their non-radiative decay, these electrons transfer energy to the surrounding medium, leading to an increase in the temperature of the colloidal solution (Fig. 2).

Graphene, a 2D material that can be also used as a PTT agent, presents good absorption,^14^ high thermal conductivity^15^ and high carrier mobility.^16^ Thus, graphene oxide (GO)^17^ and reduced graphene oxide (rGO)^18^ show a photothermal effect upon irradiation due to their broad absorption spectrum extending from the visible to near-infrared region. Furthermore, the main photothermal applications of these two graphene derivatives imply their combination with gold nanoparticles, leading to an enhanced photothermal effect.^19–23^

PTT is commonly achieved by hyperthermia of cancer cells. At a temperature below 43 °C, cells are mostly viable, between 43 °C and 49 °C, they undergo either apoptosis or necroptosis, and at temperatures above 49 °C, necrosis is the preeminent cause of cell death.^24^ Different materials are used as PTT agents, such as noble metal nanomaterials such as gold nanoparticles,^25,26^ semiconductors,^27^ carbon-based nanomaterials,^28^ and even polymers.^29^

To enhance the PTT effect on cells, heat shock proteins (HSP) should not be highly expressed. These proteins play an important role in inhibiting the thermal response of cells. Thus, by reducing their expression, the PTT effect can be highly beneficial in destroying cancer cells.^30^

The synergistic effect of PDT and PTT is an emerging scientific direction for possible application in cancer treatment. A study proved that a nanoplatform that can exhibit both PDT and PTT increased the cell death by 84% compared to 55% and 41% for PDT and PTT, separately.^31^

This review focuses on the applications of gold nanoparticle (AuNP), graphene oxide (GO), and reduced graphene oxide (rGO) nanoplatforms in photothermal therapy (PTT) for cancer treatment, with emphasis on both *in vitro* and *in vivo* studies. It provides a comprehensive overview of the various types of AuNPs and their photothermal, photodynamic and photoacoustic (PA) performances.

As two-dimensional materials with high surface areas, GO and rGO not only function as efficient PTT agents but can also serve as carriers for drugs and fluorophores, enhancing cancer treatment visualization through techniques such as fluorescence, Raman, and thermal imaging. Thus, combining the PTT effect of gold nanoparticles and graphene derivatives, herein GO and rGO, respectively, synergistic PTT effects can be achieved, as well as enhanced imaging, potentially increasing the survival rate of patients. Finally, the future prospects and limitations of hybrid nanoplatforms combining both graphene derivatives and AuNPs are presented.

## 2. Intrinsic PTT of plasmonic gold nanoparticles

**Fig. 3:**
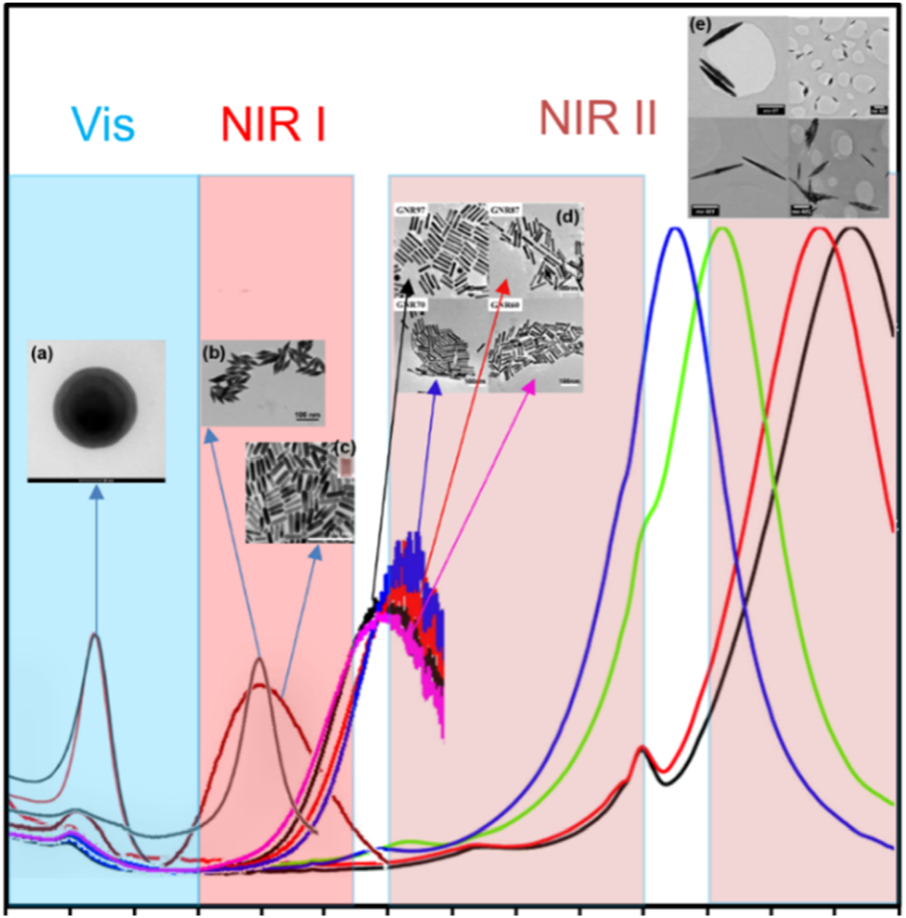
Visible and NIR spectral windows of gold nanoparticles: (a) GNSs (inset: TEM image of GNSs). Reproduced from ref. 35 with permission from Elsevier, Copyright 2021. (b) GBPs (inset: TEM image of GBPs). Reproduced from ref. 69 with permission from the American Chemical Society, Copyright 2019. (c) GNRs (inset: TEM image of GNRs). Reproduced from ref. 54 with permission from the American Chemical Society, Copyright 2013. (d) GNRs (inset: TEM image of GNRs). Reproduced from ref. 50 with permission from the American Chemical Society, Copyright 2020. (e) GNJs (inset: TEM image of GNJs). Reproduced from ref. 32 with permission from The Royal Society of Chemistry, Copyright 2015.

Noble metal nanoparticles exhibit high photothermal effects due to the presence of LSPR, which can be excited in the entire visible and near infrared (NIR) spectral window depending on the nanoparticle size and shape (Fig. 3). The NIR spectral window, mainly the first (NIR I) and second (NIR II) optical windows, is also called the biological window due to the low interference of NIR light with biological tissue.

However, when plasmons are excited with light at different wavelengths, it is commonly known that the highest temperature increase can be achieved by irradiating plasmons at their absorption maximum, which also corresponds with their resonance position. Moreover, it has been shown that a higher aspect ratio can result in greater scattering, making them more useful for scattering imaging techniques. Thus, highly anisotropic gold nanoparticles that present LSPR bands in the second NIR region (1000–1350 nm), such as GNRs, and even further gold nanojavelins^32^ (GNJs) (Fig. 3) could be used for scattering bioimaging.

### 2.1 Gold nanospheres (GNSs)

Since the first method for the synthesis of GNSs was reported,^33^ the interest in nanotechnology has increased over the years. This technique involves the reduction of gold ions from chloroauric acid with sodium citrate, resulting in GNSs with a diameter of 20 nm. Diverse methods to obtain GNSs have been investigated to modify their diameter, and thus their optical properties. For instance, Brust *et al.*^34^ successfully synthesized 1–3 nm size clusters using sodium borohydride and a two-phase mixture. To further control the size of the GNSs, Suarasan *et al.*^35^ synthesized GNSs using the seed-mediated method. Thus, 72 nm GNSs with a maximum extinction at 545 nm were synthesized. An outstanding method to obtain GNSs is using pulsed laser ablation.^36^ In this case, using a 120 fs pulsed laser (Ti/sapphire), either small GNSs (15–40 nm) or big GNSs (200–250 nm) could be synthesized, and this mixture exhibited a maximum absorption at 550 nm compared to the GNSs with a size of 3–30 nm, which had the maximum absorption of 520 nm.

To increase the biocompatibility of GNSs, different coating strategies can be used, which can change their thermal properties and induce some specific PTT. For instance, biopolymers are usually used for coating the surface of GNSs. Thus, Chirivì *et al.*^37^ synthesized GNSs coated by a protein, herein keratin, to test the photothermal performance of GNSs. It was shown that the 25 nm GNSs coated with keratin exhibited the maximum absorption at 534 nm. Furthermore, for their identification in cells, the structure of GNSs was labeled with FITC dye. The cell viability was investigated using a 3D bioprinted material and glioblastoma cells. Upon 2 min irradiation with a 532 nm laser, in resonance with the GNS extinction, the cells incubated with GNSs for 56 h showed a temperature increase of 16 °C compared to the cells incubated with GNSs for 24 h, which showed a temperature increase of 6 °C.

Compared to the study by Chirivì *et al.*,^37^ Qu *et al.*^38^ irradiated adenosine monophosphate-modified GNSs with an 808 nm laser, showing a maximum temperature increase up to 53 °C, with a power conversion efficiency of higher than 50% for laser power in the range of 1 to 2 W. The cell viability assay showed the low cytotoxicity of the GNSs, which was further investigated *in vitro* and *in vivo*. When irradiated for 10 min, the cells treated with GNSs showed high cytotoxicity. Moreover, the GNSs were introduced intratumorally for *in vivo* evaluation. After 5 min of irradiation, the temperature increased to 52.3 °C. The tumor volume did not increase, and the highest concentration of gold was found in the tumor and kidney, and thus GNSs could be excreted through the kidney.

GNSs could be also used in photoacoustic imaging (PAI) as photoacoustic (PA) agents due to their absorption. Photoacoustic imaging (PAI) merges the principles of laser-induced optical absorption with ultrasound to produce high-resolution tissue images. When tissue absorbs pulsed laser light, it experiences a slight increase in temperature, causing a rapid pressure change that generates acoustic waves. These waves travel through the tissue and are detected by ultrasound receivers at the surface. By measuring the time it takes for the sound waves to reach the detectors, an image is reconstructed.^39^

However, although the optical properties of GNSs are excellent in the visible region, it is known that in the visible region, the light is absorbed and scattered by tissue, and thus NIR region optical properties are an excellent option for lower tissue interference and better spatio-temporal resolution. Although GNSs by themselves absorb light in the visible region, their instability towards ammonia can reduce their stability in solution and tune the LSPR from the visible to NIR region. Moreover, in H_2_O_2_ medium such as the tumor microenvironment, strings of GNSs are formed with an optical response in the second NIR region, making them appropriate PA agents in the best resolution optical window.^40^

Similar aggregation techniques for better PA resolution include HOCl-responsive nanoplatforms on the surface of GNSs,^41^ pH-responsive nanosystems,^42–44^ forming bigger GNSs from ultrasmall GNSs through H_2_O_2_ responsive nanoplatform,^45^ DNA aptamer displacement of aptamer conjugated GNSs,^46^ and enzymatic cleavage of peptide conjugated GNSs.^47^ In other ways, glutathione, a mercaptan overexpressed in cancer tumors, can be used for disulfide bond cleavage in GNSs coated with polydopamine (PDA) and stabilized with PEG *via* disulfide bonds. In this case, in PBS and glutathione medium, the GNS-PDA aggregate led to better absorption in the NIR region and a better PA signal.^48^

Recently, GNSs were coordinated to form gold nanochains (GNCs), with an increased PA signal due to their NIR response. Theoretical calculations demonstrated that the absorption cross section of GNCs coated with PDA is higher than GNRs chains and pure GNRs, generating higher pulsed heat under irradiation. The efficiency of the GNCs were demonstrated *in vivo* by firstly functionalizing the PDA-coated GNCs with tripeptides to target cancer cell breast line MDA-MB231, leading to a 300% better PA signal compared to the saline-treated tumor.^49^

### 2.2 Gold nanorods (GNRs)

GNRs present two plasmon modes, a transverse plasmon in the visible range (around 530 nm) along the short axis of the GNR and a highly tunable longitudinal LSPR band from visible to NIR spectral range. The tunability of the longitudinal mode of the LSPR can be used to irradiate GNRs in resonance with a larger excitation wavelength range compared to GNSs, especially in the biotransparency window in the NIR region, which can lead to a higher increase in temperature. For example, the longitudinal LSPR of GNRs could be tuned in the second NIR window (>1000 nm), increasing their temperature to about 60 °C starting from room temperature when irradiated with a 980 nm laser with a power density of 0.5 W cm^−2^.^50^

Ooi *et al.*^51^ presented a numerical study involving a mouse model standing in three different positions, *i.e.* on its back, on its abdomen and on its side, to identify the influence of the natural convection inside the bladder. It was found that when natural convection is not considered, the tumor temperature could reach 55.7 °C. However, when the natural convection is considered, the temperature can decrease to 49.95 °C when the mouse is laying on its abdomen due to the natural convection of the bladder and the cooling of the skin with the ambient air.

One factor with a significant influence on the efficiency of PTT using GNRs is the laser power. Accordingly, Yang *et al.*^52^ proposed to study the temperature increase based on laser irradiation power using MR (magnetic resonance) thermometry. Firstly, they tested the photothermal therapy of the GNRs using a tumor phantom. Upon irradiation with a laser at 3.8 W cm^−2^, the temperature increased between 6 °C and 8 °C. Thus, to achieve a higher PTT effect, the temperature increase was measured using a 6.4 W cm^−2^ laser, resulting in a temperature increase of 14 °C and 6 °C in the surrounding area. The *in vivo* experiment was performed using a tumor phantom inserted in the thigh of a mouse. Conclusively, the GNR phantom presented a temperature increase of 26.19 °C, while the control phantom showed a lower increase of 12.64 °C, respectively. Thus, considering this high increase in temperature, and the sensitivity of the MR thermometry used in determining the temperature increase in both *in vitro* and *in vivo* studies, one can conclude that GNRs present an intrinsic PTT effect and MR thermometry can be used extensively as a PTT imaging technique.

Cancer cell death occurs through different mechanism such as apoptosis and necrosis. Moros *et al.*^53^ discovered the cellular death mechanism based on different morphologies of GNPs, namely GNRs and gold nanoprisms (GNPrs). Firstly, the functionalization of GNPs with PEG led to the better biocompatibility of GNRs and GNPrs. By irradiating the GNRs and GNPrs with a 1064 nm laser, the power conversion efficiency was 28.4% for GNPrs and 28.3% for GNRs, respectively. After that, the cellular death mechanism was investigated using apoptosis and necrosis markers. The results showed that GNPrs kill the cells through necrosis pathway. However, GNR PTT present both necrosis and apoptosis pathways.

Additionally, by synthesizing a hybrid GNR core in an Ag shell, a core–shell structure could be obtained. This structure could absorb in a wide spectral range from 300 to 1350 nm. By controlling the distance between the GNR core and Au/Ag shell, it was observed that the temperature increased by decreasing the distance between the core and the shell.^54^

Based on the surface versatility the GNRs, they can be functionalized with melanin or polymers, and then grafted with different imaging agents, such as Gd^3+^ for magnetic resonance imaging (MRI) and ^64^Cu^2+^ for positron emission tomography (PET). Nevertheless, bare GNRs could be used in PAI.^55^ Moreover, the PA properties of GNRs were combined with fluorescence imaging using IR 1061 dye and the chemo-PDT properties of an Ru-complex in a polymer-based vesicle. After NIR irradiation, the PA signal decreased due to the disassembly of the vesicle, leading to better observation of the release of the Ru-complex in tumors based on the PA signal intensity. Alternatively, the disassembly of the vesicle was observed by fluorescence imaging. Initially, the IR 1061 fluorescence was quenched in the vesicle, but after NIR irradiation, the IR 1061 dye was released and its fluorescence was recovered.^56^

Glutathione is a molecule present in the tumor microenvironment, and leveraging this characteristic, GNRs can be functionalized with two polymers at their termini, incorporating gold nanoclusters. This modification leads to a reduction in absorbance. In the presence of glutathione, the two polymers degrade, thereby releasing the gold nanorods and enhancing the PA signal *in vivo*. Upon irradiation in the second NIR region, the temperature increase was 29.4 °C.^57^

PDA could be used to coat the GNRs as a stabilizing agent during irradiation and increase their PA activity.^58^ In addition, functionalizing GNRs with peptides,^59^ antibodies,^60^ folate receptor targeting molecule,^61^ and hyaluronic acid (HA)^62^ could facilitate their efficient uptake by cancer cells.

Moreover, GNRs with longitudinal LSPR in the second NIR region could be used for PAI.^63^ Hollow GNRs are special class of GNRs that do not possess a gold core. In a recent study, Cai *et al.* synthesized miniature hollow GNRs (M-AuHNRs) with dimensions of 24.7 × 46.1 nm and a response in the second NIR region and compared them with large hollow GNRs with an extinction close to 1064 nm and a length of 105 nm. The photothermal effects were similar during irradiation; however, the PA signal was higher for M-AuHNRs compared to L-AuHNRs. The better PA properties of M-AuHNRs were further validated *in vivo*, where after 24 h of incubation, the PA signal intensity was higher than that of L-AuHNRs.^64^

### 2.3 Gold nanobipyramids (GBPs)

GBPs, an anisotropic type of plasmonic gold nanoparticles, present tunable LSPR in the NIR region, making them excellent photothermal agents for cancer treatment. Compared to GNRs, the NIR LSPR peak of GBPs is narrower. Moreover, due to their sharp tips, a stronger local electric field can be obtained at the extremities of GBPs. Moreover, their photoluminescence quantum yield is also higher than that of GNRs.^65^

**Fig. 4:**
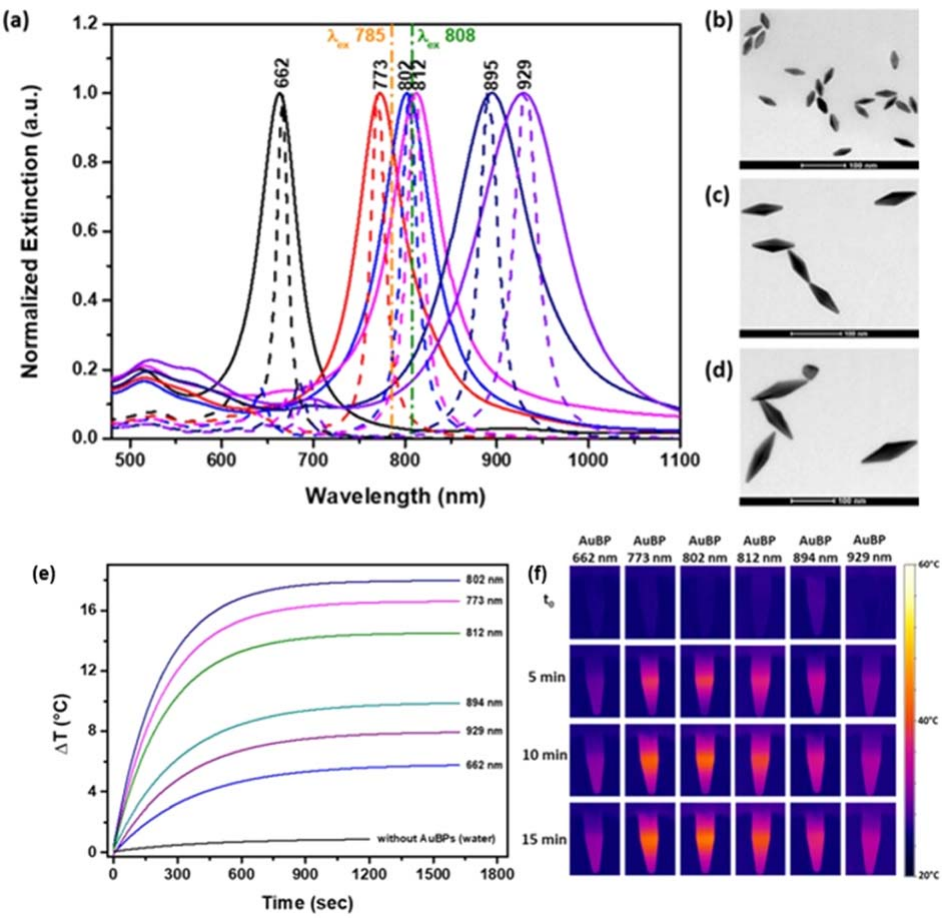
(a) Normalized experimental (continuous lines) and simulated (dotted lines) of GBPs. TEM images of GBPs with selected LSPR at 662 (b), 802 (c), and 929 nm (d). Temperature profile of GBPs irradiated with a 785 nm laser (e). Thermal camera images of GBPs irradiated with a 785 nm laser (f). Reproduced from ref. 66 with permission from IOP Publishing, Copyright 2019.

For instance, Campu *et al.*^66^ tuned the longitudinal LSPR of GBPs in the NIR region and measured their photothermal conversion efficiencies (PCE) using two different laser wavelengths of 785 and 808 nm (Fig. 4a). The morphology of the GBPs was confirmed using TEM for the GBPs with a plasmonic response at 662 nm (Fig. 4b), 802 nm (Fig. 4c) and 929 nm (Fig. 4d). Using the thermographic maps (Fig. 4f), the temperature of the probes was recorded and plotted. It was found that high PCEs were achieved when the longitudinal LSPR of the GBPs was closer to the irradiation wavelength. Thus, when irradiated with a 785 nm laser, the GBPs with a longitudinal LSPR at 802 nm had a PCE of 97%. The results also showed a high PCE of 74% for the GBPs with a longitudinal LSPR at 812 nm when irradiated with an 808 nm laser. Furthermore, the temperature increase was investigated by modifying the laser power and the volume and concentration of GBPs. It was found that the maximum increase in temperature was 19 °C for GBPs irradiated with a 785 nm laser (Fig. 4e) and 25 °C for GBPs irradiated with an 808 nm laser, thus proving the PTT effect of the GBPs.

Previously, several studies employing both PDT and PTT reported their good synergistic effects against cells. Campu *et al.*^67^ synthesized GBPs loaded with indocyanine green (ICG) to demonstrate the synergistic effect of PDT and PTT. Surprisingly, GBP showed an intrinsic PDT effect, decreasing the intensity of the 9,10-anthracenediyl-bis(methylene)dimalonic acid (ABDA) peak by 30% after irradiation at 785 nm and 32% after irradiation at 808 nm. The PTT effect increased by 2 °C when ICG was added to the GBPs, and the generation of ^1^O_2_ doubled it. The synergistic effect led to a cell death of above 90%. A similar study was conducted by Li *et al.*^68^ using GBPs coated with mesoporous silica (SiO_2_) loaded with ICG. It was found that the temperature increase was 40 °C when GBP@SiO_2_-ICG was irradiated with an 808 nm laser for 5 min, having a photothermal conversion efficiency of 33.5%. The viability test on A375 melanoma cells showed a viability higher than 90% when GBP@SiO_2_-ICG was used without irradiation. However, after irradiating the cells with an 808 nm laser for 5 min, the viability decreased to almost 20%. *In vivo* experiments were conducted on mice that were injected with A375 cells. After that, GBP@SiO_2_-ICG was introduced in the tumor site and irradiated with a laser for 5 min and the temperature increased to 46.7 °C. The survival rate of the mice treated with GBP@SiO_2_-ICG increased to more than 40 days.

Wu *et al.*^69^ investigated the PTT effect of colloidal GBPs at various concentrations with an 808 nm laser, observing an increase in the temperature by about 35 °C. They indicated that the increase in temperature was dependent on the concentration of the GBPs. Therefore, at a concentration of 30 μg mL^−1^, the temperature increased by about 32.5 °C while at a concentration of 5 μg mL^−1^, the increase was about 22.5 °C. By functionalization with a DNA strain and Texas Red, the PTT effect increased the temperature by almost 30 °C, with a photothermal conversion of 67%. The DNA strand, which possessed a fluorophore (Texas Red) and a quencher (GBPs) at both ends, was very sensitive to temperature changes. With an increase in temperature by the photothermal effect of GBPs, the distance between Texas Red and GBPs increased, leading to a higher fluorescence intensity. The live/cell imaging showed a large number of dead HeLa cells upon irradiation of the cells treated with the GBP-DNA-Texas Red nanoplatform with 808 nm laser with an intensity of 2 W cm^−3^ for 10 min.

Targeted PTT is a very important aspect of photothermal agents due to their higher accumulation at the tumor site compared to healthy tissue, thus maximizing the PTT effect. Similarly, Wu *et al.* used an aptamer, sgc8, labelled with Texas Red. The sgc8 aptamer was utilized to specifically target the PTK 7 proteins that are overexpressed in CCRF-CEM cells (human acute lymphoblastic leukemia T lymphocyte). Using a similar mechanism, when the sgc8 aptamer specifically bind to tyrosine kinase 7, an overexpressed protein on the surface of cancer cells, the distance between the fluorophore, *i.e.* Texas Red, and the quencher, *i.e.* GBPs, increased, leading to a higher fluorescence intensity from the fluorophore, thus proving the specific targeting of the GBPs to cancer cells. At a concentration of 20 μg mL^−1^, the viability of the cells was higher than 85%. After irradiating the GBPs (30 μg mL^−1^) with an 808 nm laser (1 W cm^−2^ for 10 min), the temperature increased by 25 °C. Although the PCE of the GBPs is 51.1%, the functionalized GBPs showed a decrease in PCE by only 5.6%.^70^

To achieve better biocompatibility, lipoic acid could be used in combination with PEG. Xu *et al.*^71^ found a high yield synthesis of GBPs using a dual surfactant system based on CTAB and sodium oleate (NaOL). They found that NaOL could reduce the gold from Au^3+^ to Au^0^. Thus, they synthesized GBPs with a maximum absorption at 915 nm and irradiated them with a 915 nm laser. Prior to irradiation, the GBPs were functionalized with PEG and lipoic acid. The increase in the temperature for the functionalized GBPs was about 53 °C after the first irradiation for 3 min with a 915 nm laser 0.8 W cm^−2^. The *in vitro* studies showed the high viability of 4T1 murine breast cells when the functionalized GBPs were introduced without irradiation. However, when irradiated with the same laser at a power of 1 W cm^−2^, the viability of the cells decreased to 5%. Considering the good PTT effect in the *in vitro* studies, they verified the PTT *in vivo*. The temperature increased to 52 °C for the *in vivo* studies after irradiation with a 915 nm laser with an intensity of 0.8 W cm^−2^ for 5 min. The survival rate of the mice injected with the functionalized GBPs was 100% after 30 days.

Moreover, GBPs could be used for chemo-photothermal therapy by loading DOX on the PDA-functionalized surface of GBPs. Due to the addition of PDA and DOX, the temperature increment of the GBPs increased from 15.8 °C to 25.8 °C in the tumor, where the chemo-photothermal nanoplatform was used for both photothermal imaging and PAI.^72^

### 2.4 Gold nanotriangles (GNTs)

Gold nanotriangles (GNTs) present wider LSPR peaks, which red-shift with the length of their tips.^73^ GNTs are usually prepared using seed-mediated methods. Interestingly, halide ions could be used for the anisotropic growth of GNTs. However, by modifying the ratio of gold nanoseeds and iodide ions, the LSPR bands of GNTs could be tuned between 630 nm and 750 nm.^74^

**Fig. 5:**
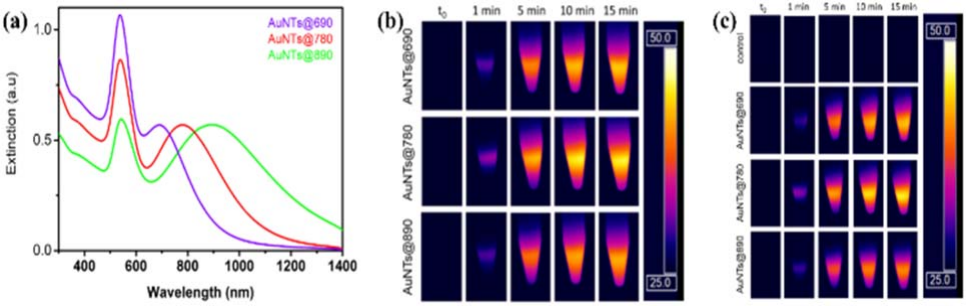
(a) Extinction spectra of 3 different types of synthesized gelatin-GNTs. (b) Thermal camera images of the GNTs in solution irradiated with 785 nm laser (b) and in biological phantoms (c). Reproduced from ref. 75, Copyright 2022.

Prior to using photothermal agents for *in vivo* studies, it is recommended that the nanoparticles provide a PTT effect in similar biological-mimicking tissue. Thus, Suarasan *et al.*^75^ synthesized GNTs using a gelatin biopolymer as a reducing and stabilizing agent. Three GNT samples with the maximum extinction of 690 nm, 780 nm and 890 nm were successfully synthesized (Fig. 5a). Upon irradiation with a 785 nm laser for 15 min, the highest temperature increase of 22 °C was achieved with the 780 nm GNTs (Fig. 5b). Following that, they tested the GNTs on a biological phantom simulating biological tissue. Accordingly, the highest temperature increase was found in the case of the 780 nm GNTs of 21 °C (Fig. 5c). Thus, the GNTs were subsequently used for *in vitro* PTT in B16.F10 murine melanoma cells. The viability of the cells treated with GNTs with no irradiation was above 80%. However, when irradiated with a 785 nm laser at 196 mW for 15 min, the lowest cell viability was 55% for the 780 nm GNTs.

Janus nanoparticles are a class of hybrid asymmetric nanoparticles combining two different natures on both sides of the nanoparticles. For instance, they could provide PTT using one side of their surface and drug delivery system their other surface. Thus, Wang *et al.*^76^ synthesized Janus GNTs with mesoporous silica, folic acid (FA) and tirapazamine for combined PTT and chemotherapy. For PTT application, a 980 nm laser 0.5 W cm^−2^ was used for 3 min, while radiotherapy was achieved using X-ray radiation with 1 Gy min^−1^. The *in vitro* results showed the effect of the GNTs on different cell lines, such as hypoxic cells and normoxic cells (SMMC-7721 and HL-7702). In the case of both hypoxic and normoxic cells, combining both irradiation methods resulted in lower cell viability. The *in vivo* results were evaluated using 8 groups of mice bearing SMMC-7721 tumor cells. By using FA as a targeting agent in tandem with NIR and X-ray irradiation of the GNTs, the authors concluded that the tumor inhibition was higher compared to non-specific targeted GNTs.

### 2.5 Gold nanostars (GNSts)

GNSts, one of the most anisotropic gold nanoparticles, present multiple tips, which exhibit a high electromagnetic field enhancement, making them good candidates as photothermal agents. Also, these nanoparticles could be reshaped post-synthesis.^77^

A good description of the photothermal effect of GNSts could be achieved by irradiating them with a laser. Usually, GNSts present a wide FWHM (full width at half maximum) due to the irregularities in their spikes such as length and width, thus presenting wide absorption throughout the visible and NIR spectral range. To quantify the wide absorption range of the GNSts, Gherman *et al.*^78^ irradiated small and large GNSts with a 785 nm laser. Small GNSs, which have an absorption maximum at 531 nm, were irradiated off-resonance. Similarly, small GNSts with an extinction maximum at 614 nm were also irradiated off-resonance with respect to their longitudinal plasmonic band. In contrast, large GNSts with an extinction maximum at 730 nm were irradiated close to their resonance. As expected, the temperature increase profile confirmed that the closer to the resonance irradiation occurs, the higher the increase in temperature.

To provide better control of the size and shape, D'Hollander *et al.*^79^ synthesized GNSts with a size of 67 nm by modulating the HAuCl_4_ addition rate using a syringe pump. Effective *in vitro* internalization of the GNSts was visualized using CT (computed tomography) and PAI (photoacoustic imaging) in SKOV3 ovarian carcinoma cells. The PTT *in vitro* was demonstrated by irradiating with a 690 nm laser (7 W cm^−2^), resulting in a temperature increase of 25 °C. The *in vivo* PTT was measured on tumor-bearing mice using a laser power of 2 W cm^−2^ for 5 min. After irradiation, the volume of the tumor decreased to almost 50% after 15 days compared to the control tumor, the size of which increased to about 184% after 8 days.

To efficiently target cancer cells, polydopamine (PDA) can be successfully used. Furthermore, studies showed that dopamine (DA) could also increase the blood flow.^80^ In this context, Li *et al.*^81^ synthesized GNSts functionalized with PDA and PEG. The highest temperature increase was achieved with an 808 nm laser (2 W cm^−2^) for an irradiation time of 10 min. To test the *in vitro* PTT, the biocompatibility of GNSts was tested using the CCK-8 test. The viability of the HeLa cells was greater than 85% when the concentration was 460 μg mL^−1^. Thus, the PTT effect was demonstrated on HeLa cells using diverse irradiation power intensities from an 808 nm laser. Showing the best PTT, the 2 W cm^−2^ intensity was used to measure the PTT effect with time. Thus, after 5 min, the viability of the cells decreased to 10%.

**Fig. 6:**
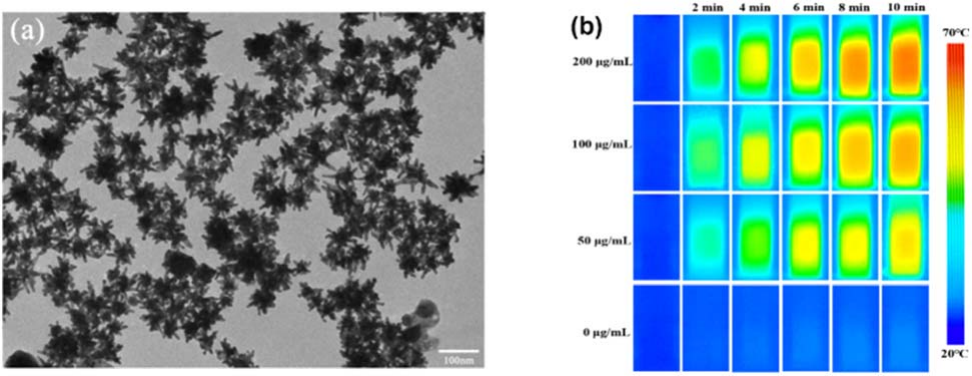
(a) TEM image of FA-GNSts. (b) Photothermal images of FA-GNSts at different concentrations irradiated with an 808 nm laser. Reproduced from ref. 82 with permission from the American Chemical Society, Copyright 2021.

Hu *et al.*^82^ synthesized GNSts, as confirmed by TEM (Fig. 6a), which were functionalized with FA for both PTT and CT. The biocompatibility of the synthesized GNSts was investigated using the CCK-8 test, which showed the high viability of the cells of above 90%. The efficiency of the GNSts as therapeutic agents was tested for both PTT and radiation therapy (RT). In the case of PTT, an 808 nm laser (2 W cm^−2^) was used, while RT implied an X-ray dose of 6 Gy. Notably, a concentration-dependent increase in temperature was observed when FA-GNSts were irradiated (Fig. 6b), presenting a PCE of 28.12%. The results showed a higher decrease in cell viability when RT was used compared to laser irradiation. However, when used simultaneously, a synergistic effect was observed.

Moreover, Zhang *et al.*^83^ synthesized a site-specific targeted nanoplatform based on GNSts as a PTT agent covered with a mesoporous silicon shell functionalized with chlorin e6 (Ce6). The novelty of this research is based on functionalization with catalase, which was used to produce oxygen from oxygenated water to enhance the PDT. Furthermore, they functionalized it with a phospholipid to target tumors. The biocompatibility was tested on MCF-7 cells and HeLa cells. The results showed the higher cytotoxicity of the nanoplatform on HeLa cells due to its higher targeting of these cells. It was demonstrated that the targeting nanoplatform had a PTT effect on HeLa cells compared to the non-targeting nanoplatform due to its higher cellular uptake. The PDT effect was investigated under a 660 nm laser. When the cells were irradiated with a 660 nm laser, there was a low decrease in the cell viability. However, when the cells were irradiated with both an 808 nm laser (for PTT) and 660 nm laser (for PDT), the killing effect was much more efficient. The *in vivo* results showed a high temperature increase to more than 60 °C at a concentration of 24 mg mL^−1^. The volume of the tumor in the tumor-bearing mice was also small when the nanoplatform was injected and irradiated with both laser wavelengths.

In terms of the PAI and fluorescence imaging techniques based on GNSts, Li *et al.*^84^ coated GNSts with PDA and grafted them with the Ce6 fluorophore. This nanoplatform not only exhibited excellent photothermal properties and ^1^O_2_ generation, but also showed increased photothermal stability for efficient PAI *in vivo*.

One of the biggest challenges in terms of PAI using anisotropic GNPs is their photothermal stability. Usually, the heat generated upon the irradiation of pure anisotropic GNPs can lead to morphology changes due to laser-induced morphological phenomena. However, new strategies emerged in the case of GNSts to retain their shape over a longer duration of irradiation by coating them with silica shells.^85^ Taking a step further, silica-coated GNSts were functionalized with ICG and embedded in mesenchymal stem cells. The stem cells were proven to deliver nanoparticles in tumors.^86^ Grafting ICG on the nanoplatform was performed for fluorescence visualization of the stem cells. The role of plasmonic nanoparticles in the nanoplatform is for PAI and PTT.^87^ Furthermore, by using Prussian blue in combination with GNSts to create Prussian blue-coated GNSts, the PA signal of the GNSts could also be enhanced in MRI.^88^

### 2.6 Gold nanoshells (GNShs)

Gold nanoshells (GNShs) are usually obtained through the seed-mediated method. By modulating the ratio between the core diameter and shell thickness, the LSPR response could be tuned even to the NIR spectral region.

Kalinowska *et al.*^89^ synthesized hollow GNShs using a thiolated aptamer for efficient binding to epithelial cancer cells. The temperature increase was measured after irradiation with an 808 nm laser for 2 min. The maximum temperature achieved was about 100 °C. Selective binding to the tumor cells was assessed using four tumor cell lines including A549 and MCF-7 that overexpress the MUC1 protein and MRC-5 and MCF-10A that exhibit low MUC1 protein. Accordingly, it was found that the cellular uptake of GNShs was higher for the cell lines that overexpress the MUC1 protein. Notably, it was found that after two doses of irradiation, the cell viability decreased to 59% for A549 cells, similar to the MCF-7 cells, which represent the cell lines that overexpress the MUC1 protein.

For improved biocompatibility towards hepatic cancer cells, Ma *et al.*^90^ synthesized GNShs on silica nanorattles with thiolated PEG and verified the photothermal effect *in vitro* and *in vivo*. The morphology of the GNShs was confirmed by TEM, while the optical characterization was determined using UV-Vis spectroscopy. The temperature of the GNShs was registered using a thermal camera that acquires thermal images. After 5 min of irradiation with an 808 nm laser at 1 W cm^−2^, the temperature increased by 17 °C. After irradiating HepG2 cells treated with PEGylated GNShs, the cell viability decreased to 18.3%. The *in vivo* PTT effect was investigated in rabbits treated with the abovementioned GNShs *via* intraarterial administration (i.a.) and intravenous administration (i.v.). However, it was seen that the tumor volume decreased when the GNShs were i.a. and irradiated with an 808 nm laser (2 W) for 3 min.

Moreover, Yang *et al.*^91^ synthesized GNShs with a cisplatin-loaded human serum albumin (HSA) core for chemo-PTT. Upon irradiation with an 808 nm laser (1.5 W cm^−2^) for 10 min, the temperature of the synthesized nanoplatform increased by 29.1 °C, proving that they are suitable for PTT. The viability of the A549 cancer cells treated with the chemo-photothermal nanoplatform and irradiated with 1.5 W cm^−2^ laser decreased to less than 10% when the concentration of cisplatin was above 50 μM. The *in vivo* experiments were evaluated in mice. The HSA-GNShs alone showed a temperature increase in the tumor of more than 10 °C, which demonstrates the potential of the GNShs as PTT agents. Nevertheless, the cisplatin-loaded HSA-GNShs presented a higher temperature increase, and also some tumor clearance.

Bian *et al.*^92^ synthesized anisotropic GNShs showing “spike-like” forms on the surface. Upon irradiation with an 808 nm laser at a power of 1.5 W cm^−2^, the nanostructure solution increased to 72.6 °C after 5 min. The PTT effect *in vitro* was confirmed by irradiating HeLa cells treated with GNShs with a laser, leading to a low cell viability as the power of the laser and the concentration of the GNShs increased. The *in vivo* PTT effect was measured using the same laser with a power density of 1.5 W cm^−2^ for 5 min on a U14 cell-induced tumor in mice. The tumor temperature increased to 54.8 °C, which correlates with a lower tumor volume due to the photothermal therapy, effectively ablating cancer cells.

Wang *et al.*^93^ synthesized silica-core GNShs with a chromophore based on palladium, which were used as a PS for both PDT and PTT. However, the PDT effect of the PS-modified GNShs was lower compared to the free PS under irradiation with an LED at a wavelength of 550 nm, which was used to facilitate only ROS measurement. Moreover, when irradiated with an 800 nm pulsed light, a higher generation of ROS was demonstrated compared to LED illumination. They further demonstrated that the functionalized GNShs could induce DNA damage when irradiated *via* the expression level of γ-H2AX, a marker for DNA harm. The *in vitro* PDT/PTT effect was demonstrated by irradiating the treated cells with an 800 nm pulsed laser with a power of 1 W cm^−2^ for 5 min, showing low cell viability, which was slightly above 20%.

Moreover, silica-based GNShs with a diameter of less than 100 nm exhibited a better absorption coefficient compared to larger GNShs, making them suitable PTT and PAI agents.^94^ Similarly, nanoscale metal organic frameworks coated with GNShs present PTT and PAI but in the second NIR region.^95^

## 3. Graphene derivatives as PTT and PDT agents

### 3.1 GO as intrinsic PTT agent

GO is a two-dimensional nanomaterial derived from graphene, which has oxygen-containing functional groups on its surface such as epoxide, carbonyl, carboxyl, and hydroxyl groups. Unlike graphene, which is hydrophobic, GO is hydrophilic, which results in better water dispersibility.^96^ GO has been widely explored as a PTT agent for cancer therapy because it can generate heat when irradiated with light.

In a recent study, Liang *et al.*^97^ used a GO concentration of 100 μg mL^−1^ in water and an 808 nm laser with a power density of 1.5 W cm^−2^, irradiating for 10 min, and achieved a temperature increase of 40.6 °C. Moreover, by functionalizing GO with FA, they achieved a targeting photothermal agent for PTT. Subsequently, the 808 nm laser was used to determine the influence of the laser power on the photothermal effect. It was observed that by increasing the power density from 1 to 2.5 W cm^−2^, the maximum temperature also increased from approximately 45 °C to almost 90 °C. Further, they investigated the concentration effect on the temperature increase by FA-GO during irradiation with an 808 nm laser (1.5 W cm^−2^) for 10 min. By irradiating an FA-GO solution with a concentration of 20 μg mL^−1^, the temperature increase was almost 15 °C, while for a 5-fold higher concentration, the temperature increase was almost 40 °C. This indicates that both the concentration and laser power have a strong influence on increasing the temperature of the probe.

However, other studies have found that even at lower concentrations and laser powers, significant temperature increases can still be achieved. For example, Ma *et al.*^98^ used a concentration of only 10 μg mL^−1^ and a laser power of 0.5 W cm^−2^ at 808 nm, irradiating for 10 min, and observed a temperature increase of 21.2 °C. Paradoxically, Ma *et al.*^98^ obtained better results using a lower concentration and a lower laser power than Guo *et al.*,^99^ who used a 2 W cm^−2^ laser power for 10 min and a GO concentration of 25 μg mL^−1^, and the solution temperature increased by 17 °C.

Interestingly, the choice of laser wavelength also appears to have an impact on the photothermal effect by GO. For instance, Liu *et al.*^100^ used a laser wavelength of 980 nm and a power of 0.5 W cm^−2^ at a concentration of 50 μg mL^−1^ and irradiation time of 10 min, resulting in a temperature increase of around 23 °C. This indicates that even at low concentrations and laser power, a high temperature increase can still be achieved by using an appropriate wavelength.

Taken together, these studies demonstrate the potential of GO as a highly efficient photothermal agent for a variety of applications, including cancer therapy and drug administration. However, researchers need to look closely at the various factors, such as the level of oxidation in GO, the laser power and potential photothermal enhancers such as polymers, that can influence the photothermal effect and optimize these parameters to reach the desired level of temperature increase. As research in this field continues to progress, it is likely that GO and other nanomaterials will play an increasingly important role in the development of new photothermal therapies and diagnostic techniques.

GO is a very cell-friendly substance because it has none or very low toxicity, even at a high concentration. Zhang *et al.*^101^ showed that for HeLa cells, the cell viability for an aqueous solution of GO remained over 90% in the concentration range of 1.25 μg mL^−1^ to 40 μg mL^−1^. These results remained unchanged even when the probes were irradiated for 1 min using an 808 nm, 2 W cm^−2^ laser without GO. The same cell viability was achieved by Liang *et al.*,^97^ even when they increased the concentration from 10 to 200 μg mL^−1^.

Mostly, in the literature, researchers used low GO concentrations in the cell viability test, but interestingly, Guo *et al.*^102^ exposed the cells to a larger GO concentration range of 4 μg mL^−1^ to 20 mg mL^−1^. The results were consistent with the previous studies shown before. In the case of concentrations lower than 125 μg mL^−1^, the cell viability was over 50%, while for concentrations of 250 μg mL^−1^ to 20 mg mL^−1^, the cell viability decreased to under 50%. As expected, the cell viability decreased with an increase in the concentration of GO, which resulted in cell death.

Moreover, photoacoustic imaging (PAI) is an imaging modality that has gained popularity due to its deep penetration and great spatial resolution. Jun *et al.*^103^ synthesized a chitosan-folic acid-GO nanoplatform and confirmed its PTT application as well as bioimaging by both fluorescence imaging and PAI. Using different wavelengths, the accumulation of the nanoplatform at the tumor site was confirmed after 24 h, demonstrating the long-lasting blood circulation of the nanoplatform. The same circulation time was observed in fluorescence imaging using IR-783 as a contrasting agent. GO has a low NIR absorption compared to rGO. Thus, to further increase it, grafting fluorophores on its surface can lead to better optical properties in the NIR window. Thus, by using FA and ICG, two major challenges can be solved. FA can efficiently target cancer cells, while ICG is used as both a photothermal agent and imaging agent. Therefore, nanoplatforms consisting of FA, ICG and GO are suitable for PTT and as PAI agents.^104^ In addition, GO can also enhance the fluorescence of dyes. This phenomenon is attributed to the fact that between GO and a porphyrin-derived dye, the PEG polymer could facilitate charge transfer. Moreover, this nanoplatform could be used for both fluorescence (due to its high intensity) and PAI.^105^

Due to their excellent loading capacity, mesoporous silica nanoparticles (MSNs) could be used in combination with GO to achieve a higher concentration of dyes on the surface of GO nanoplatforms, without affecting the PA signal intensively. Thus, two-photon imaging techniques, as well as PAI could be achieved using a single imaging agent.^106^

### 3.2 rGO as intrinsic PTT agent

rGO is a derivative of GO that has been partially restored to the original structure of graphene, which is a single layer of carbon atoms arranged in a hexagonal lattice.^107^ rGO has some advantages over GO such as high stability, large surface area, tunable optical properties, and easy functionalization. rGO can be used as a PTT agent for cancer therapy because it can absorb NIR light and convert it into heat, which can destroy tumor cells. rGO can also be functionalized with different materials, but it can also be used as an intrinsic PTT agent. In fact, the photothermal effect of rGO is influenced by a variety of factors, including concentration, laser wavelength, laser power, and irradiation time. In particular, higher rGO concentrations, longer irradiation times, and higher laser powers tend to result in greater temperature differences. Overall, these findings suggest that the photothermal effect of rGO can be optimized by carefully controlling these experimental parameters, and further research in this area can lead to the development of novel applications for this material in a range of fields.

Several studies have explored the photothermal effect of rGO, examining how different experimental conditions affect the resulting temperature differences. The studies varied in the laser wavelength, power, and irradiation time, as well as rGO concentration used. The wavelengths used were 790 nm ^108^ and 808 nm,^109^ with irradiation times ranging from the minimum of 180 s ^110^ to the maximum of 600 s.^109^ The rGO concentrations varied from 10 μg mL^−1^^109^ to 1.2 mg mL^−1^.^108^ Notably, even if the concentration was not very high, the laser power and irradiation time have a great impact. Therefore, a high increase in temperature of above 50 °C was obtained by Chiu *et al.*^111^ using a laser power density of 2.72 W cm^−2^ for 300 s at a concentration of 0.3 mg mL^−1^ of rGO-iron oxide-hydroxide. However, at a higher rGO concentration, better results can be obtained, even if the laser power is lower. For example, Amina *et al.*^108^ achieved an increase in temperature of around 60 °C using a concentration of 1.2 mg mL^−1^ rGO with a 790 nm laser at a power density of 2 W cm^−2^. Also, slightly large temperature differences could be obtained even at a low rGO concentration when increasing the irradiation time. Irradiating the probe for 600 s with an 808 nm laser (1 W cm^−2^) at a concentration varying from 2.5 μg mL^−1^ to 10 μg mL^−1^, Zhang *et al.*^109^ achieved an increase in temperature of 9.4 °C and 22.2 °C, respectively. Moreover, by irradiating rGO functionalized with a photosensitizer based on Ru(ii), the yield of ^1^O_2_ generated was 0.06 when the nanoplatform was irradiated with a 450 nm laser for 8 min.

Overall, these results indicate that the photothermal effect of rGO is strongly influenced by the specific experimental conditions used, and thus further investigation is needed to determine the optimal conditions for this effect.

Chen *et al.*^112^ synthesized an imaging nanoplatform of PEGylated rGO and ICG to test its bioimaging properties *in vivo*. Firstly, using an agarose phantom, it was observed that the PA signal of the GO-based platform was lower than that of the rGO-based platform due to the better loading efficiency of ICG on rGO, and also the higher absorption intensity of rGO. Moreover, the *in vivo* fluorescence imaging showed the tumor accumulation of the rGO-based imaging agent after 48 h. In addition, PAI was further used for in-depth localization of the nanoplatform inside the tumor region with a very high resolution. The fact that the degree of reduction on rGO and its absorption play an important role in the increase of PA was demonstrated previously.^113^ Similarly, Sheng *et al.*^114^ presented the same advantage of using rGO for PAI both in phantoms and *in vivo*. The use of dyes in fluorescence and PAI is an excellent option to gather significant results on the tumor location. By combining these two imaging techniques, better resolution can be achieved *in vivo*. It was demonstrated that nanoplatforms based on rGO and dyes have a higher PA signal compared to rGO.^115^

Further research showed that rGO-based nanoplatforms with PDA, mesoporous nanoparticles and DOX could be used for chemophotothermal therapy and as PA agents.^116^ PDA-coated rGO was used with ICG to increase the PA signal. It was observed that ICG-PDA-rGO presented a 20-fold stronger PA signal compared to GO. However, the *in vivo* experiments showed that ICG-PDA-rGO has a PA signal 4-times higher than GO.^117^

In summary, both GO and rGO have found extensive utility in various therapeutic modalities, such as PTT and PDT. A widely used approach involves the functionalization of GO and rGO with diverse proteins, polymers, and drugs including PEG, FA, PVP, ICG, methylene blue (MB), hyaluronic acid (HA), chitosan (Chit), and doxorubicin (DOX), aimed at increasing their therapeutic efficacy. Table 1 presents a comprehensive overview of the pivotal GO and rGO-based nanomaterials found in the recent literature utilized for PTT and PDT applications, including key insights into the radiation sources and the experimental focus, covering *in vitro* and *in vivo* studies.

**Table 1:** Summary of gold nanoparticle and reduced graphene oxide-based hybrid materials for PTT and PDT applications in cancer treatment

Composition	Method	Radiation source	*In vitro*	*In vivo*	Ref.
**Based on GO**					
GO-PEG	PTT	808 nm	RAW274.7 macrophage HOS cells	Osteosarcoma	118
MnO_2_-FA-GO	PTT	808 nm	HeLa cells	—	119
FA-GO-ICG-DOX	PTT	808 nm	HeLa cells	Cervical cancer	120
FA-CS-GO	PTT	808 nm	MDA-MB-231	Triple-negative breast cancer	103
mGO-CS/SA	PTT	808 nm	A549 cells	—	121
MCGO–HNPa	PDT	700 nm	HepG-2	—	122
GO-PEG (TP)	PTT, PDT	980 nm	4T1 cells	Breast cancer	100
PEG-FA-GO	PTT, PDT	808 nm		Adenocarcinoma	123
GO-FA/Ce6	PTT, PDT	808 nm	MCF-7 cells	—	99
		660 nm			
ICG-Wed-GO	PTT, PDT	808 nm	HeLa cells	Carcinoma	101
GO-MB/PF127	PTT, PDT	660 nm	SiHa cells	—	98
		808 nm			
PTX@GO-PEG-OSA	PTT, PDT	808 nm	HGC-27 cells	Gastric carcinoma cell line	102
GO-PEG	PTT, PDT	808 nm	4T1 cells	Breast cancer	124
GO-Pd	PTT, PDT	808 nm	PC3 cells	Prostate cancer	125
ICG-GPP	PTT, PDT	808 nm	MCF-7 cells	—	126
NCGO-MB-FA	PTT, PDT	660 nm	HeLa and MCF-7 cells	—	
		808 nm			97

**Based on rGO**					
HA-rGO	PTT	808 nm	MCF-7 cells	—	127
Green-Tea-rGO	PTT	780 nm	HT29, SW48 cells	—	128
FNP/rGO-PEG	PTT	805 nm	4T1 cells	Breast cancer	129
RES + DOPA-rGO@Gel	PTT	808 nm	MCF-7 cells	—	130
CMC-rGO/CHO-PEG	PTT	808 nm	L-929 cells	—	131
P-DOPA-rGO (PEOX)	PTT	808 nm	MCF-7 cells	—	132
rGO-PAH/DOX	PTT	808 nm	MCF-7 cells	—	133
rGO-MSN-PDA	PTT	808 nm	MHCC97L, MHCC97H cells	—	134
PCS-rGO (PEG + Chit)	PTT	808 nm	MCF-7 cells	—	135
PPIX-rGO	PDT	635 nm	HeLa, HADF cells	—	136
rGO-THPPEG	PTT, PDT	550 nm	HeLa cells	—	137
		808 nm			
rGO-MnO_2_-PEG-UCNP-Ce6	PTT, PDT	808 nm	HeLa cells	Carcinoma	138
rGO-Ru-PEG	PTT, PDT	808 nm	A549 cells	Lung carcinoma	139
		450 nm			
rGO-MnO_2_	PTT, PDT	808 nm	HeLa cells	—	140
FeOxH-rGO	PTT, PDT	808 nm	T47D, 4T1	Breast cancer	111
Chit-rGO-IR820-DOX	PTT, PDT	785 nm	C26 cells	—	141
rGO-HA/DOX	PTT, PDT	808 nm	U87 cancer cells, 3T3 fibroblast cells	Gliomas	110
GPC3-modified rGO-PEG	PTT, PDT	808 nm	HepG2 cells	—	142

## 4. PTT and PDT applications of graphene-gold nanoparticle hybrids

As presented above, gold nanoparticles as well as graphene derivatives present reliable properties for both PDT and PTT. Thus, there has been increasing interest in designing hybrid nanoplatforms, consisting of gold nanoparticles and GO and rGO. For example, by combining the PTT properties of gold nanoparticles with the high surface area and chemical properties of GO and rGO, novel cancer therapy platforms can be obtained. GO and rGO-based nanoplatforms are usually obtained through electrostatic interactions due to the difference in charge between GNPs and graphene derivatives. Other ways to obtain similar nanoplatforms are by functionalizing GNPs with fluorophores, aptamers or organic substances that present aromatic rings. In this way, π–π stacking between graphene derivatives and functionalized gold nanoparticles is the preeminent interaction that leads to the generation of hybrid nanoplatforms.

### 4.1 Gold nanospheres and graphene oxide nanoplatforms (GNSs-GO)

One of the biggest concerns regarding GNSs-GO nanosystems is that GNSs are not deposited in a high amount on GO sheets. Thus, to overcome this, the *in situ* generation of GNSs on GO using sodium citrate was proposed. Firstly, GO was treated with sodium hydroxide (NaOH) and chloroacetic acid (ClCH_2_COOH) to increase the amount of carboxylic moieties (–COOH) on GO. The carboxylic (–COOH) groups present on graphene oxide (GO), along with the –COOH groups of sodium citrate, coordinate with the [AuCl_4_]^−^ ions. The deprotonated carboxylic groups of citric acid nucleate the gold seeds and complex the AuCl_4−*x*_OH_*x*_ derivatives obtained in the aqueous solution of HAuCl_4_, leading to the rapid growth of the seeds on the surface of GO. Furthermore, the reaction temperature plays an important role in obtaining monodisperse GNSs. Then, the GNSs could be deposited on carboxylated GO. To improve the dispersability of the nanocomposite in water, PEG-SH was used.

By irradiating GNS-GO-PEG with a 532 nm laser at a power density of 21 W cm^−2^, the temperature increased by 20 °C. However, when GNS-GO-PEG was irradiated with an NIR laser (*λ* = 808 nm) at a power density of 29 W cm^−2^, the temperature increased by 17.3 °C. The cytotoxicity was determined on a normal epithelial colon cell line (HCEC-1CT) and gastric carcinoma cell line (N-87). Notably, GNS-GO showed very low dark cytotoxicity towards cancer cells, but moderate cytotoxicity in the normal epithelial colon cell line.^143^

Wang *et al.*^144^ synthesized eggshell membrane (ESM) with GO and GNSs in two different ways. The first method involved the *in situ* generation of GNSs on the eggshell membrane by mixing HAuCl_4_ and eggshell membrane at 95 °C. After that, the eggshell membrane and GNS (GNS/ESM) composite was immersed in a GO solution to obtain GO/GNSs/ESM. The second way involved the immersion of ESM in a GO solution, followed by the introduction of GO/ESM in HAuCl_4_ and heat treatment to obtain GNSs/GO/ESM. This intriguing method showed that the eggshell membrane could reduce the gold ions. The PTT effect was examined by irradiating the platform with simulated sunlight using a xenon lamp. By irradiating the eggshell membrane, the temperature increased by 10.2 °C. However, when the ESM was functionalized with GNSs and GO (GNSs/GO/ESM), the temperature increased to 47.3 °C, while with GO and GNSs (GO/GNSs/ESM) the temperature increased to 49 °C, showing an improved PTT efficiency compared to pristine ESM and proving its potential application in PTT.

He *et al.*^145^ synthesized GO/SiO_2_ layer/GNSs using a seed-mediated method. Firstly, gold seeds were obtained, followed by their addition to the GO/SiO_2_ nanostructure. Then, the complex of seed-decorated GO/SiO_2_ was introduced in the growth solution. The *in vitro* photothermal activity of the hybrid nanoplatform showed that the photothermal effect of GO/SiO_2_/GNSs irradiated with an 808 nm laser (0.3 W cm^−2^, 20 min) decreased the cell viability to 10%, while the temperature increased by 11.7 °C.

To increase the electrostatic interactions between GNSs and GO, there should be distinct charges on GNSs and GO. Thus, GNSs could be coated with a negatively charged polymer, and inversely GO could be functionalized with a positively charged polymer to change its charge. Upon irradiation with an 808 nm laser (500 mW), the temperature increased by about 7 °C. In comparison, when GO was functionalized with IR780 dye, the temperature increased by 7.5 °C. This temperature further translated into a higher PCE. Thus, for GNSs-GO, the PCE was 22.01%, while that for GO-IR780 was 22.75%. When cancer cells were treated with the hybrid nanoplatform (GNSs-GO) and irradiated with an 808 nm laser, the viability decreased to below 20%.^146^

Considering the need to actively target cancer tissues for selective PTT, Yang *et al.*^147^ synthesized a GNS-GO nanoplatform conjugated with MUC1 aptamer for selectively targeting breast cancer cells. The temperature increase by the MUC1-GNS-GO system was higher compared to that of MUC1-GNSs and GO, exceeding 15 °C when irradiated with an 808 nm laser for 5 min with a power of 3 W. The *in vitro* PTT showed an increase in temperature to up to 53 °C after irradiation for 5 min. The selectivity of the nanoplatform showed preferential binding to breast cancer cells. Thus, by irradiating the cells containing the nanoplatform, the cell viability decreased to 57%. In a similar manner to that reported by Yang *et al.*,^147^ Chauhan *et al.*^148^ synthesized a targeted nanoplatform for both PTT and chemotherapy using FA as an active targeting compound for folate receptors that are overexpressed in most cancer cells. DOX was used as the drug model and was loaded on FA-GNS-GO through electrostatic interaction. Next, the release of Dox from the nanoplatform was observed to be influenced by both the pH and NIR irradiation conditions. The temperature increase by irradiating the nanosystem with an 808 nm laser for 5 min was higher than that by GO, FA-GO and Dox. The synergistic photochemotherapy effect of DOX-FA-GNS-GO was observed, leading to less than 20% viability in both HeLa and MCF-7 cancer cells. It should be noted that not only PTT and chemotherapy cause cell death but also the generation of ROS, *i.e.* superoxide and hydrogen peroxide generation. The in *vivo* antitumor efficacy presented a high percentage survival (70%) in the DOX-FA-GNS-GO-treated mice when irradiated with a laser. However, DOX alone resulted in a survival rate of just about 40%.

The thermal effect of GO/GNSs was investigated using the heat shock protein (HSP) and caspase 3 gene (CASP3), which are indicators for apoptotic pathways in cells. Upon irradiation with an 808 nm laser (1.2 W cm^−2^), the synergistic thermal effect of GO/GNS was enhanced, with a temperature increased of 15 °C compared to the bare GNSs and GO (5 °C and 7 °C, respectively). Furthermore, the expression level of HSP increased when the nanohybrid platform was irradiated. This fact demonstrates that the DNA repair mechanism could be suppressed by the NIR laser irradiation of the synthesized platform. Not only HSP overexpression was found to be induced by irradiation of GO/GNSs, but also the CASP3 genes were overexpressed. Thus, cancer cell apoptosis could be achieved.^149^

**Fig. 7:**
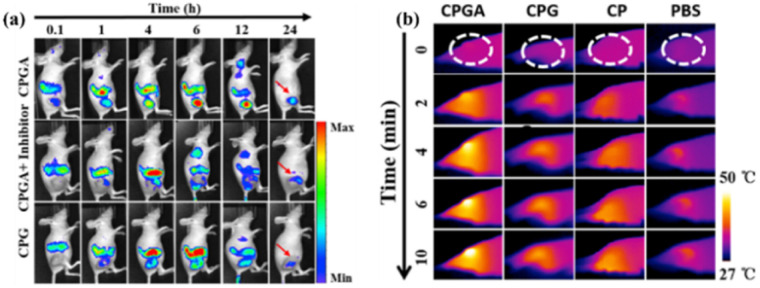
(a) *In vivo* fluorescence imaging of tumor-bearing mice treated with CPGA. (b) Thermal images of tumor-bearing mice treated with the nanoplatform and irradiated with 808 nm laser for 10 min. Reproduced from ref. 150 with permission from Elsevier, Copyright 2016.

For real-time imaging-guided PTT, a complex nanoplatform based on GO and GNSs functionalized with Cy5.5 in a metalloproteinase-14 matrix was obtained. This nanoplatform exhibited high fluorescence when metalloproteinase-14 was degraded. By irradiating with an 808 nm laser for 10 min, the temperature increased by 23 °C, showing strong NIR absorption and decreasing the viability of the cells by 80%. When administered to tumor-bearing mice, the tumor to muscle ratio showed high values. By using the fluorescent properties of the nanoplatform, *in vivo* NIR fluorescence was used to identify the tumor location. Notably, after 24 h, the fluorescence intensity was concentrated at the tumor site (Fig. 7a). Moreover, by irradiating the tumor treated with the nanoplatform, *i.e.* CPGA, thermal images showed a higher temperature increase at the nanoplatform location, indicating once again high accumulation at the tumor site (Fig. 7b). *In vivo* studies showed that the temperature of the irradiated area increased by 15.9 °C, the relative tumor volume reduced and no recurrence after 14 days.^150^

### 4.2 Gold nanospheres and reduced graphene oxide nanoplatforms (GNSs-rGO)

Dual modality cancer therapy can enhance the therapy effect of nanomedicines by sensitizing cancer cells to chemotherapeutic drugs. Given that GNS-rGO nanoplatforms can be functionalized with targeting compounds such as FA, dopamine (DA) and hyaluronic acid (HA), the overall systemic toxicity of drugs can be reduced and concentrated in tumors. Mirza-Aghayan *et al.*^151^ proposed a precise method for the drug release of DOX in the nanohybrid platform rGO/DA/GNSs/DOX, by including DA for targeting purely cancer cells. Firstly, they obtained rGO/DA using the well-known EDC/NHS method. Further, they obtained GNSs *via* the reduction of HAuCl_4_ with sodium citrate. The rGO/DA complex was added to a GNS solution for the synthesis of rGO/DA/GNSs, which was further mixed with a DOX solution to obtain the rGO/DA/GNS/DOX hybrid. The photothermal properties of the hybrid were investigated using a sun simulator with a power of 0.1 W cm^−2^ and 0.3 W cm^−2^. Expectedly, the highest temperature was 47.3 °C after irradiation for 5 min at the power of 0.3 W cm^−2^. The DOX release was found to be exceptionally better in an acidic medium. Similarly, Qi *et al.*^152^ constructed a hybrid nanoplatform with GNRs/GO@PDA using DOX as the model drug. The increase in the temperature by GNRs/GO@PDA was 28 °C when irradiated for 60 min with an 808 nm laser at a power of 2 W cm^−2^. This result may be due to the synergistic thermal effect of the GNR/GO@PDA platform. The pH responsiveness of drug release was measured for GNRs/GO@PDA with DOX. Higher drug release was measured in an acidic medium, which was further triggered by an NIR laser.

Ma *et al.*^153^ improved the biocompatibility of a rGO/gold nanocluster (rGO/GNC) platform with a modified polymer consisting of PEG and 3-(3-phenylureido) propanoic acid (PPA) to form PPEG. According to the cell viability tested *in vitro* on HeLa cells, the hybrid nanoplatform exhibited no cytotoxicity on cells without irradiation. The photothermal effect was measured using an 808 nm laser at different intensities and various concentrations of rGO/GNCs/PPEG. Thus, at a 90 μg mL^−1^ concentration in aqueous solution, the temperature increased to 54.8 °C when irradiated for 5 min with a power intensity of 3 W cm^−2^. This platform was loaded with DOX to provide both PTT and chemotherapy. It was proven that upon irradiation, the rGO/GNC/PPEG/DOX platform exhibited better cell cytotoxicity than using bare DOX or rGO/GNCs/PPEG.

Given that both rGO and GNSs present light absorption capacity, a nanoplatform consisting of rGO and Cys conjugated GNSs could be used for efficient PTT as well as PAI. Interestingly, using a laser source in the second NIR region, the PA signal was increased for the nanoplatform compared to rGO and GNSs alone.^154^

### 4.3 Gold nanorods and reduced graphene oxide nanoplatforms (GNRs-rGO)

Yu *et al.*^155^ proposed a method to obtain GNRs/rGO *via* femtosecond laser ablation. They found that during the irradiation, the longitudinal and the transversal planes of the GNRs decreased, leading to higher aspect ratios. To compare the photothermal conversion efficiency, they measured the temperature variations in different solutions including water alone, GNRs, rGO and GNRs/rGO. Subsequently, it was found that the photothermal conversion of GNR/rGO was from 60.4% up to 77.8% depending on the duration of irradiation from an 808 nm, 0.5 W laser, compared to rGO (56.4%) and GNRs (38.8%).

Compared to the well-known reducing agents for GO, such as hydrazine and ascorbic acid, Zhang *et al.*^20^ obtained rGO-GNRs using l-cysteine as a reducing agent for GO. The cell viability test showed low dark cytotoxicity even after 48 h at a concentration of 300 μg mL^−1^. However, after irradiating the cells containing rGO-GNRs for 15 min with an 808 nm laser at 2 W cm^−2^, the cell viability decreased to 22% for a concentration of 300 μg mL^−1^.

An interesting drug delivery system consisting of rGO and GNRs was proposed by Yang *et al.*^156^ They used hydroxyapatite on the rGO/GNR nanoplatform as s pH-responsive carrier. The PTT effect was evaluated using an 808 nm laser with a power intensity of 1 W cm^−2^. When irradiated for 10 min, the hydroxyapatite nanoplatform showed a lower temperature increase compared to the rGO-GNR dispersion. However, when the drug delivery nanosystem containing 5-fluorouracil (5-FU) was irradiated, the cell viability assay showed a higher death percentage compared to the rGO-GNR nanoplatform. By adding hydroxyapatite to rGO-GNR, the biocompatibility of the newly synthesized nanoplatform increased.

Jaswal *et al.*^157^ synthesized a GNR-rGO@PCL platform and tested its photothermal activity in breast cancer ablation as well as nerve regeneration in tissue. In fact, polycaprolactone (PCL) is an FDA-approved polymer that possesses high tensile strength and can be used in tissue engineering. After 5 min irradiation with an 808 nm laser, 90% of the MCF-7 cancer cells were dead when the hybrid platform had the highest concentration (O.D. 4, 0.00068 wt% PCL) of GNRs-rGO, while for O.D. 2, O.D. 1 and free PCL, the viability of the cells decreased by 75%, 48% and 19.8%, respectively. The temperature increased up to 47.6 °C after irradiation of GNRs-rGO@PCL for 5 min at 0.72 W cm^−2^ with an 808 nm laser. To verify the nerve regeneration, PC-12 and S-42 (Schwann cell line) cells were incubated in a medium that contained PCL and rGO-GNR platform with the above-mentioned concentrations. It was observed that after 7 days, the proliferation of the cells was high when rGO-GNRs was mixed with PCL at the highest concentration and under NIR irradiation. It was suggested that the hot electrons generated from the irradiation of the GNRs were carried by the rGO lattice, which might induce electrical stimulation of nerve cells, thus leading to high cell proliferation. The aligned scaffolds of PCL were used for cell growth guidance. Moreover, it was also proven that graphene maintained cells proliferation and encouraged the development of neural stem cells, while the combination between graphene and GNPs was used for PTT.

**Fig. 8:**
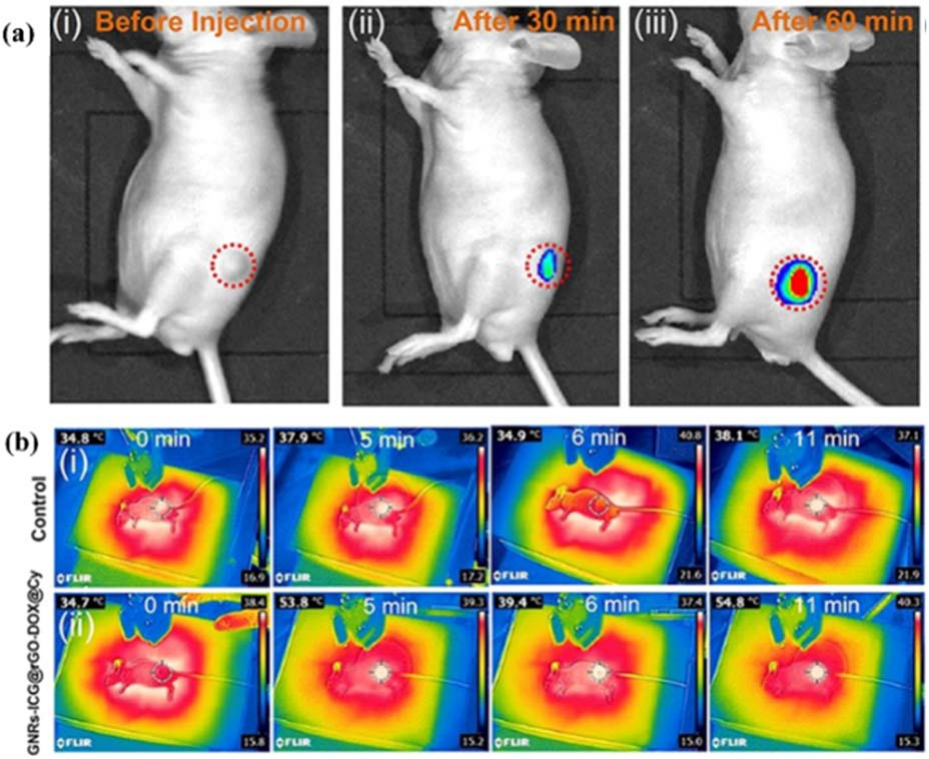
(a) *In vivo* fluorescence imaging of mouse treated with GNRs-ICG@rGO-DOX@Cy-5.5. (b) Thermal camera images of mice treated with (i) PBS and (ii) GNRs-ICG@rGO-DOX@Cy-5.5 and irradiated with 808 nm laser (2 W cm^−2^). Reproduced from ref. 158, Copyright 2022.

A multimodal cancer therapy nanoplatform based on GNRs coated with mesoporous silica, indocyanine green (ICG) fluorophores and rGO and DOX drug was prepared by Maji *et al.*^158^ It was observed that the enhanced photothermal property of the plasmonic nanoparticle coated with mesoporous silica together with the use of rGO, DOX and ICG presented a higher temperature increase compared with pure ICG and GNRs with ICG. High toxicity towards HT-29 cancer cells was observed for GNRs-ICG@rGO-DOX under NIR irradiation at 808 nm, 2 W cm^−2^ for 10 min. Furthermore, for use as a bioimaging agent, an NIR fluorophore, *i.e.* Cy-5.5, was functionalized on the chemophotothermal agent. Thus, *in vivo* fluorescence images were obtained. As can be observed in Fig. 8a, the internalization of the nanoplatform at the tumor site was observed after 60 min. Moreover, by irradiating the mice with an 808 nm laser for 11 min, thermal camera images were acquired (Fig. 8b). As observed, the mice that were not treated with the nanoplatform did not exhibit an increase in the tumor site temperature (Fig. 8b(i)). However, when the mice were treated with the nanoplatform and irradiated with an 808 nm laser, the tumor temperature increased by up to 20 °C (Fig. 8b(ii)).

Similar to Maji *et al.*,^158^ Zhang *et al.*^159^ synthesized a drug nanocarrier based on GNRs, mesoporous silica and rGO. The drug model used was DOX. It was found that the thickness of the silica shell influenced the stability of GNRs/rGO upon irradiation. Thus, it was observed that the thinner the silica layer, the better the photothermal stability of the nanoplatform. The release of DOX drug upon irradiation was concludingly higher for the thin mesoporous silica-coated GNRs/rGO compared to the thick silica-coated GNRs/rGO. The drug release was highly influenced by the intensity power of the laser. By irradiating the GNRs coated with SiO_2_, the release of DOX was lower compared to the nanoplatform.

The selectivity of an rGO-GNR nanoplatform was achieved through an interesting approach employing the Tat protein to specifically interact with U87MG cells. The biocompatibility of the nanocomposite was higher than that of the GNRs. The PTT effect was measured using an 808 nm laser at a power of 1 W cm^−2^ on U87MG cells for 10 min. After irradiation, the cell viability decreased by 97%. The *in vivo* PTT application of the nanoplatform showed a decrease in the tumor volume in 15 days, and most of the nanoplatform was found in the tumor and blood vessels of the tumor.^160^

### 4.4 Gold nanorods and graphene oxide (GNRs-GO)

GNR-GO nanoplatforms have attracted more interest as PTT agents compared to bare CTAB-GNRs because of their higher photothermal stability under more irradiation cycles.^161^

The general PTT application of GNRs/GO was determined by Younis *et al.*,^162^ who synthesized GNRs/GO-PEG, proving PTT for HeLa cells. The photothermal conversion efficiency (PCE) reached 72% for the synthesized nanohybrid, and thus it was significantly higher than GNRs (35%) and GO (18%) tested separately. In dark conditions, the hybrid did not present cytotoxicity. However, after irradiation with an 808 nm laser at 0.3 W, the viability of the cancer cells decreased to 4% at a concentration of 0.1 mg mL^−1^. Moreover, Sun *et al.*^163^ produced GO-GNRs coated with PSS (polystyrene sulfonate) and PDDAC (poly(diallyl ammonium chloride)) to reduce the toxicity of GO-GNRs, and also proved it as a good candidate for computed tomography imaging. Upon irradiation with an 808 nm laser (0.4 W cm^−2^), the temperature increased by 24.9 °C. The *in vitro* cell viability test showed low dark cytotoxicity even at a concentration of 200 μg mL^−1^. When irradiated with the same laser with a power of 0.8 W cm^−2^ for 10 min, the viability of SW1990 cells decreased to less than 20%. Following that, the *in vivo* PTT was tested by irradiating the tumor exposed to the nanoplatform. The tumor temperature increased from 24.8 °C to 59.7 °C, and the mice healed within 15 days.

Seo *et al.*^164^ synthesized GNRs coated with mesoporous silica shell functionalized with methylene blue and GO to induce thermal stress in the cancer cells through both PDT and PTT. This study showed that the mixture of GO and GNRs in this nanoplatform induced significant thermal stress in the cells, reaching up to 52 °C when irradiated with a 785 nm laser for 25 min. When the PDT/PTT nanoplatform was introduced in MDA-MB-231 cells and irradiated with a 785 nm laser at a power density of 0.8 W cm^−2^, the viability decreased to less than 10%. Similarly, Lebepe *et al.*^165^ synthesized a hybrid platform including GO/GNRs/porphyrin as both PTT and PDT agents. Separately, the GO/GNR platform modified with PVP showed a temperature increase by 21.8 °C when irradiated with 500 mW at 785 nm for 10 min. This result makes the platform suitable for PTT due to the hyperthermic stress that can be induced in cancer cells. Regarding the singlet oxygen generation, 1,3-diphenylisobenzofuran (DPBF) was used to identify the quality of the synthesized hybrid platform. Thus, by measuring the decrease in the absorption intensity of the DPBF peak, the singlet oxygen quantum yield was found to be higher in the case of GO/GNRs/porphyrin than pure porphyrin.

Graphene-based materials also present good drug carrier characteristics. Qi *et al.*^166^ produced GNR/SiO_2_/GO-PEG to quantify the thermal properties of GNRs and test the drug delivery of DOX under NIR irradiation. It was found that the combination of GO-PEG with GNRs/SiO_2_ induced a higher temperature increase compared to bare GNRs/SiO_2_. Moreover, the stability of GNR/SiO_2_/GO-PEG was exceptional in acetate buffer solution (pH = 4.5). The DOX release was measured at pH = 4.5 under 2 W cm^−2^ irradiation at 808 nm. This study showed that the drug release was 28.72% in acetate buffer solution compared to 3.59% in deionized water and 3.05% in PBS. The PCE of the nanoplatform was 39.53%. Similarly, DOX loading was achieved by Xu *et al.*,^167^ who synthesized GNRs-GO with HA for chemo-photothermal therapy using DOX as the model drug. The excellent PTT effect showed an increase in the solution temperature to 60 °C when irradiated with an 808 nm laser for 3 min. In the absence of irradiation, the release of DOX was highly dependent on the pH of the solution, showing higher drug release at lower pH. However, when the nanocarrier was irradiated for 30 min, the drug release in 24 h increased to 34.5% compared to the non-irradiated nanoplatform (9.8%). The *in vitro* results showed low cell viability (<10%) for cells incubated with the nanocarrier when irradiated with an 808 nm laser at a power intensity of 2 W cm^−2^ for 5 min. Another drug that can also be used for chemotherapy is paclitaxel. Azerbaijan *et al.*^168^ synthesized a polymer nanofiber core with paclitaxel and GO-GNRs coated with a chitosan shell. The nanofibers exhibited low dark cytotoxicity. However, when irradiated with an 808 nm laser (1 W cm^−2^) the viability of A549 lung cancer cells decreased to below 30%. The *in vivo* chemo-PTT showed a decrease in the tumor volume of a mouse bearing A549 demonstrating tumor growth inhibition of the nanofibers.

### 4.5 Graphene and gold nanoshells

Considering the light to heat conversion properties of GNShs and excellent conductivity of graphene, a theoretical study simulated the heat conversion properties of silica-core GNShs wrapped in graphene. Notably, by irradiating spherical, prolate and oblate GNShs with an 808 nm (0.3 W cm^−2^) laser, the highest temperature increase was registered for the oblate structure (46.1 °C).^169^

GNShs with an iron oxide core deposited on rGO presented interesting results for PTT and chemotherapy. Irradiation supports both the photothermal effect by increasing the temperature up to 30 °C, as well as DOX release. The cytotoxicity of the nanoplatform was investigated using the MTT assay with HeLa cells, showing a linear decrease with an increase in the concentration of rGO. Next, by irradiating the treated cells with an 808 nm laser for 5 min, the viability of the cells decreased to less than 5%.^170^

### 4.6 Graphene and gold nanostars

Branched GNSts are of high importance in photothermal and sensing applications due to their anisotropic geometry. However, when embedded in graphene, their performances are enhanced. For example, the *in situ* synthesis of GNSts on GO decreased the aggregation of the ligand-free GNSts, as well as improving their biocompatibility, leading to a cell viability of above 90%.^22^ Interestingly, when the GO-GNSt-treated cells were irradiated with an 808 nm laser for 2 min, their viability decreased to 19% ± 2% after 24 h and complete cell death was observed after 48 h.

In addition, a nanoplatform based on GNSts and rGO coated with PDA could successfully be used as a drug carrier for DOX. As expected, high drug release was observed under 655 nm irradiation, thus reducing the cell viability to less than 10%.^171^ The *in vivo* investigation showed that the nanosystem could increase the temperature in the tumor site to 55 °C, leading to reduced tumor volumes.

The rGO/GNSts could also be used as a gene carrier for gene therapy and PTT application. Thus, to further improve the biocompatibility of rGO/GNSts, a lipid bilayer (liposome) that encapsulates the nanoplatform functionalized with FA was used. A mutant anticancer gene plasmid, KrasI, was used for gene therapy. Next, the photothermal effect was measured using an 808 nm laser, and the temperature of rGO/GNSts increased by 41.3 °C, achieving a PCE of 66.3%. The synergistic PTT and gene therapy effect was observed after 48 h from irradiation, reducing the cell viability to 14.3%, mostly by apoptosis. *In vivo* studies showed that the highest tumor growth inhibition (98.5%) was achieved through the synergistic photothermal effect of the nanoplatform and the tumor volume decreased after 14 days.^23^

Multimodal therapy of cancer presents higher efficiency compared to using only one type of therapy due to its potential synergistic effect. Thus, GO-GNSts with an absorption maximum at 660 nm were conjugated with Ce6, a fluorophore capable of producing ^1^O_2_, for a single wavelength irradiation. For better biocompatibility, PEG was used. When the GO/GNSt/PEG complex was irradiated, a temperature increase of about 50 °C was achieved in 8 min after irradiation with a 660 nm laser (2 W cm^−2^). Notably, by functionalization of the nanoplatform with Ce6, the difference in temperature increase was negligible compared to the non-functionalized nanoplatform. The singlet oxygen generation was measured using SOSG (singlet oxygen sensor green) fluorescence intensity. Ce6 showed higher singlet oxygen generation compared to GO/GNSts/PEG/Ce6, but this could be overcome by the intracellular delivery of the nanoplatform. Under dark conditions, the hybrid theranostic platform exhibited low cytotoxicity, but when irradiated with a 660 nm laser, the viability decreased to 20.7%. *In vivo* photothermal imaging and dual therapy showed a high temperature increase in the tumor and moderate temperature increase in the surrounding healthy tissue leading to a decrease in the tumor size.^172^

Moreover, the rGO-GNSt nanocomplexes could be used for both PTT and gene therapy by functionalizing the surface of the hybrid structure with the mutant anticancer K-Ras gene plasmid. Further, due to the synergistic effect, the nanosystem could achieve a tumor growth inhibition of 98.5%. In addition, taking advantage of the optical properties of rGO and GNSts, PAI was achieved with a high contrast after 24 h.^23^

## 5. Perspectives

**Fig. 9:**
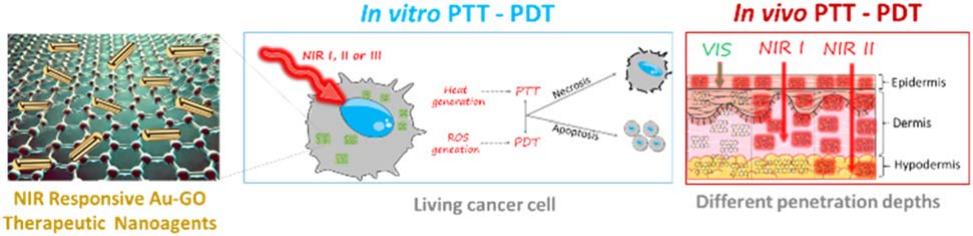
Schematic of the possible cellular death mechanisms induced by PDT and PTT in the NIR region, together with different penetration depths, depending on the irradiation wavelength.

A more detailed understanding of the cellular death mechanisms is crucial for further elucidating the therapeutic potential of various graphene-plasmonic nanoplatforms designed for the selective killing of cancer cells of interest. To date, it has been shown that the synergistic effects of PTT and PDT significantly reduce the viability of cancer cells. Moreover, the use of single-wavelength irradiation could simultaneously activate both PTT and PDT *via* the various designed graphene@plasmonic nanoplatforms. Additionally, leveraging the well-known high loading capacity of graphene can next enable the integration of multimodal therapeutic strategies, such as drug delivery, into these activable nanoplatforms. However, considering that the near-infrared (NIR) region extends beyond wavelengths of 1000 nm, future studies exploring PTT and PDT in the second NIR window (>1000 nm) can improve tissue penetration, leading to more effective and deeper tumor destruction, as schematically illustrated in Fig. 9.

Additionally, graphene-gold nanoparticle-based nanoplatforms present a broader extinction spectrum compared to free gold nanoparticles, making LEDs a promising irradiation source. These LEDs could offer a more cost-effective and flexible alternative to the currently used lasers. Lastly, due to their excellent biocompatibility and ability to significantly reduce cancer cell viability upon laser irradiation, graphene-based nanoplatforms hold great potential for future translation into preclinical trials. To date, to the best of our knowledge, only three clinical trials involving GNPs have been conducted, all of which focus on the drug delivery capabilities of colloidal gold.

The first study, Phase I and Pharmacokinetic Studies of CYT-6091, a Novel PEGylated Colloidal Gold-rhTNF Nanomedicine (NCT00356980),^173^ was a Phase I clinical trial evaluating the safety, tolerability, and optimal dosing of tumor necrosis factor (TNF)-bound colloidal gold (CYT-6091) in patients with solid tumors. The nanomedicine formulation consisted of gold nanoparticles (27 nm in diameter) functionalized with recombinant human TNF-alpha and thiolated polyethylene glycol (PEG). The results indicated that doses ranging from 60 μg m^−2^ to 600 μg m^−2^ were well tolerated, demonstrating the potential of CYT-6091 for further clinical development.

The Phase 0 Clinical Study (NCT03020017)^174^ investigated a novel nanoplatform for glioblastoma treatment. This approach utilized GNPs conjugated with RNA nucleotides designed to target the Bcl2L12 protein, a key regulator of apoptosis in glioblastoma cells. Conducted in a group of eight patients, this trial assessed the safety and biodistribution of microdose (0.04 mg kg^−1^) administrations, which were well tolerated, paving the way for the further exploration of RNA-based nanotherapies.

Finally, the EE-ASI-1 Trial (2016–2019)^175^ was conducted between 2016 and 2019 and focused on immunotherapy for type 1 diabetes. This trial investigated a therapeutic platform in which human proinsulin peptide C19-A3 was conjugated to ultrasmall gold nanoparticles (C19-A3-GNP) to modulate autoimmune responses and prevent the destruction of insulin-producing beta cells by T lymphocytes. The results demonstrated that the nanoplatform was well tolerated, suggesting its potential for antigen-specific immunotherapy in autoimmune diseases.

However, hybrid nanoplatforms based on graphene and GNPs are not used in preclinical trials due to the inconsistency of the graphene layer sizes. Moreover, a clear synthesis method to induce oxygen containing groups on GO at a specific carbon to oxygen ratio remains a challenge. Oxygen-containing groups play an important role in the functionalization of GO with biocompatible polymers and cancer cell-targeting compounds. Thus, GO may not be readily present at tumor sites, leading to potential triggering unwanted immune responses. Furthermore, rGO is not a convenient graphene derivative for phototherapies due to its poor stability in water or biological fluids, which may cause aggregation, reducing its phototherapy effect. Even if its surface could be biofunctionalized for better bioaccumulation, the electrostatic interactions could be broken inside the body over a longer period of time, and without a consistent clearance pathway, the potential damage to organs over time cannot be estimated.

## Data availability

No primary research results, software or code have been included, and no new data were generated or analysed as part of this review.

## Author contributions

Alexandru Holca: writing – original draft, review & editing; Vlad Cucuiet: writing – original draft, review & editing; Simion Astilean: writing – review & editing, Marc Lamy de la Chapelle: writing – review & editing, project administration, funding acquisition, Monica Focsan: writing – review & editing, project administration, conceptualization.

## Conflicts of interest

There are no conflicts to declare.
